# Ultra-selective ligand-driven separation of strategic actinides

**DOI:** 10.1038/s41467-019-10240-x

**Published:** 2019-06-04

**Authors:** Gauthier J.-P. Deblonde, Abel Ricano, Rebecca J. Abergel

**Affiliations:** 1 Chemical Sciences Division, Lawrence Berkeley National Laboratory, Berkeley, CA 94720 USA; 2 Department of Nuclear Engineering, University of California, Berkeley, CA 94720 USA

**Keywords:** Inorganic chemistry, Nuclear chemistry

## Abstract

Metal ion separations are critical to numerous fields, including nuclear medicine, waste recycling, space exploration, and fundamental research. Nonetheless, operational conditions and performance are limited, imposing compromises between recovery, purity, and cost. Siderophore-inspired ligands show unprecedented charge-based selectivity and compatibility with harsh industry conditions, affording excellent separation efficiency, robustness and process control. Here, we successfully demonstrate a general separation strategy on three distinct systems, for Ac, Pu, and Bk purification. Separation factors (SF) obtained with model compound 3,4,3-LI(1,2-HOPO) are orders of magnitude higher than with any other ligand currently employed: 10^6^ between Ac and relevant metal impurities, and over 10^8^ for redox-free Pu purification against uranyl ions and trivalent actinides or fission products. Finally, a one-step separation method (SF > 3 × 10^6^ and radiopurity > 99.999%) enables the isolation of Bk from adjacent actinides and fission products. The proposed approach offers a paradigm change for the production of strategic elements.

## Abstract (web summary)

Radionuclides are of great importance for fields such as nuclear medicine and waste recycling, but their efficient purification remains a challenge. Here the authors show that an octadentate hydroxypyridinone chelator enables efficient and robust separation processes for isotopes of Ac, Pu, and Bk.

## Introduction

Isotope production has been the cornerstone of many research fields and applications throughout the last century^1^ and relies largely on separation science. Contemporary examples illustrating the primary importance of separations include radionuclide purification for use in radiopharmaceuticals^2^ or in radioactive thermoelectric generators that are vital to space exploration^3^. The development of efficient separation methods is also critical for forensics analysis, recycling of ageing weapon materials, fabrication of nuclear fuels^4^, production of radiotracers for research^5^, as well as manufacturing of high-purity actinide targets for the discovery of new elements^6^. Regardless of the application, product purity must be as high as possible, which requires highly efficient and cost-effective separation methods. Radionuclide production through either target irradiation (^225^Ac, ^177^Lu, ^90/86^Y, ^89^Zr, ^47/44^Sc, ^238^Pu, ^248^Cm, ^249^Bk, ^249/252^Cf, etc.) or milking from long-lived sources (^227^Ac → ^227^Th → ^223^Ra, ^241^Pu → ^241^Am, ^233^U → ^229^Th → ^225^Ra → ^225^Ac, ^232^Th → ^212^Pb, etc.) involves the handling of mixtures of metal ions where major impurities are often neighboring elements in the periodic table. In most cases, the ratio between the valuable element and impurities is extremely high (a few μg diluted in multi-g targets), rendering purification very challenging, albeit critical for the availability of the coveted isotope.

Recent reports have highlighted the creative use of solid matrices, such as crystalline selenites^7^ or borates^8^, to quantitatively separate f-elements. However, most at-scale chemical purifications rely on chromatographic separations or liquid-liquid extraction methods or both, depending on the process scale and the desired specifications. These bi-phasic techniques are based on intrinsic interactions between metal ions and organic molecules dissolved in an organic diluent (liquid-liquid extraction) or grafted onto a solid matrix (chromatography). Ideally, these organic molecules (hereafter referred to as extractants) are amenable to transfer ions of interest from one phase to another in a selective manner relative to impurities. Extractant performance is typically not predictable and not always transposable from one separation system to another. In fact, predicting the efficiency and metal selectivity of a given process formulation (aqueous phase, diluent, and extractant) is a scientific challenge with numerous correlated variables^9,10^, such as metal speciation in the aqueous phase, metal-extractant compound speciation in the organic phase, free extractant speciation, influence of the diluent, loading capacity of the organic phase, etc. Most extractants currently used in hydrometallurgy can potentially co-extract multiple elements depending on chemical conditions (acidity, extractant concentration, phase ratio etc.)^11–13^. Separation selectivity is only achieved by finely tuning those chemical conditions and operational conditions for these processes are generally highly constrained, with many required steps to reach desired purities.

To overcome these challenges, a class of hydroxypyridinone (HOPO) chelators was investigated for its high metal-binding selectivity and applicability to separation needs. These molecules exhibit a unique combination of properties long sought in separation science: (i) water-solubility, (ii) structures consisting of solely H, C, N, and O atoms, (iii) ability to control metal oxidation states without additional redox-active species, (iv) extremely high charge-based selectivity, and (v) high metal-ligand complex stability even in strong acid (up to 10 M H^+^). Using the model octadentate HOPO chelator, 3,4,3-LI(1,2-HOPO) (hereafter referred to as 343HOPO), and taking advantage of these unprecedented characteristics, highly efficient and robust chemical separation processes were developed for three strategic examples: the purification of ^225^Ac, Pu-isotopes, and ^249^Bk.

## Results and Discussion

### Choice of aqueous chelator as hold-back reagent

**Fig. 1:**
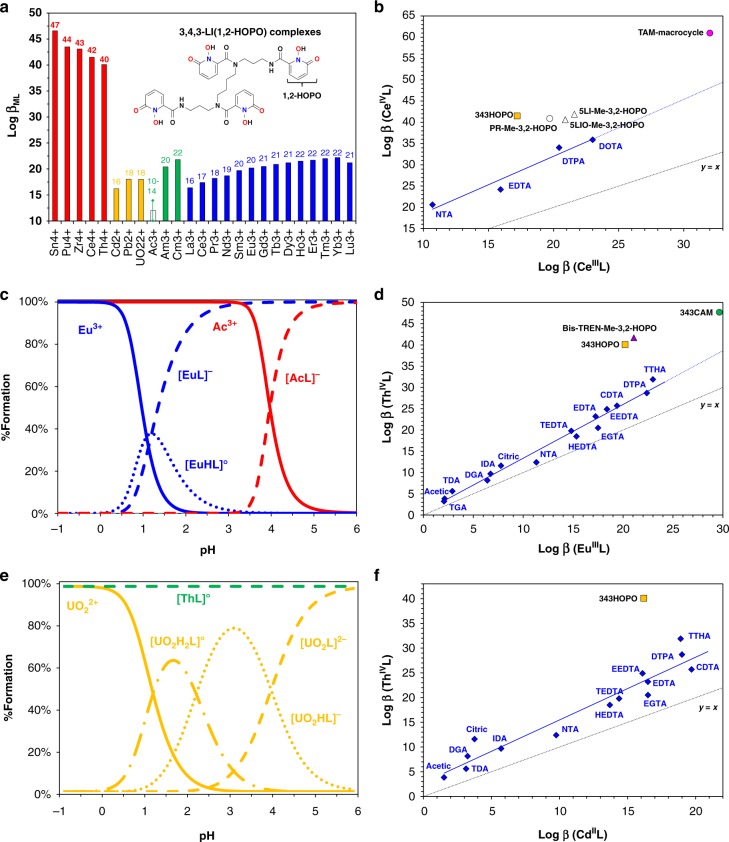
Relative thermodynamic stabilities of metal-ligand complexes discussed in this work. **a** Stability constants (log *β*_ML_)^24,25,46,48–50^ of 343HOPO complexes with tetravalent cations (red), divalent ions (yellow), trivalent actinides (green), and trivalent lanthanides (blue). The value plotted for Ac^3+^ is an estimate based on its ionic radius. Selectivity comparisons for Ce^4+^/Ce^3+^ (**b**), Th^4+^/Eu^3+^ (**d**), and Th^4+^/Cd^2+^ (**f**), with HOPO and CAM ligands and classical chelators used in separations. For tetradentate ligands 5-LIO-Me-3,2-HOPO and 5-LI-Me-3,2-HOPO, the β_ML2_ value is used. For bidentate PR-Me-3,2-HOPO, the β_ML4_ value is used. The line y = x corresponds to no selectivity. Additional selectivity comparisons are given in Supplementary Fig. 2. Full ligand names and structures are given in Supplementary Table 1. Metal speciation diagrams for 343HOPO solutions containing Ac^3+^ or Eu^3+^ (**c**) and UO_2_^2+^ or Th^4+^ (**e**). [Chelator]/[Metal] = 1 mol/mol. L = Ligand. Solid lines: free metal. Dotted lines: 343HOPO complexes

Numerous drug development studies^14–16^ have focused on synthetic siderophore-inspired compounds because of their ability to form stable, and sometimes luminescent, complexes with metal ions of interest for medical imaging, radionuclide decontamination, and cancer treatments. While this class of ligands, encompassing HOPO and catecholamide (CAM) derivatives, has been known for decades, it had never been studied in details for separation applications. In fact, a few exceptions aside, most of these chelators have only been studied with a single cation, such as Gd^3+^, Th^4+^, or Pu^4+^, impairing broader evaluation of their metal-metal selectivity. The chemistry of some HOPO ligands was recently extended across the periodic table, highlighting their outstanding selectivity, and large superiority over polyaminocarboxylate chelators (IDA, EDTA, DTPA, etc.), typically encountered in separations (Fig. 1). A comparison of metal complex formation constants for the octadentate 343HOPO shows striking differences between tetravalent species and corresponding divalent or trivalent ones. The observed selectivity seems mainly charge-based and only slightly dependent on the ionic radius of the cations (Supplementary Fig. 1), although some selectivity is noted across the trivalent lanthanide and actinide series. 343HOPO outperforms any known chelator in terms of charge-specific selectivity and, in particular, for the binding of tetravalent ions. As such, the Ce^4+^/Ce^3+^ selectivity of 343HOPO is about 15 orders of magnitude higher than what could be expected from a carboxylate ligand. Extreme selectivity is also observed even for ions of similar size, such as in the Th^4+^/Am^3+^, Th^4+^/Gd^3+^, Ce^4+^/Lu^3+^, or Th^4+^/Cd^2+^ pairs (Fig. 1 and Supplementary Fig. 2). As shown in Fig. 1, a handful of other molecules from the same family (octadentate 3,4,3-LI-CAM, Bis-TREN-Me-3,2-HOPO, and TAM-macrocycle; tetradentate 5LI-Me-3,2-HOPO, 5LIO-Me-3,2-HOPO; and bidentate PR-Me-3,2-HOPO)^17–20^ exhibit known solution thermodynamic properties that match those of 343HOPO but none of the chelators typically used for separation methods is nearly as selective. Many additional HOPO and CAM ligands have been designed^15^ and previously reported octadentate 1,2-HOPO^21^, mixed 3,4,3-LI(1,2-HOPO/Me-3,2-HOPO)^22^, and DFO/1,2-HOPO^23^ structures would certainly make excellent candidates for charge-based selectivity; however, their chelation properties have so far only been investigated with either Eu^3+^, Zr^4+^, or Pu^4+^. Due to its current kg-scale availability and established solution thermodynamics, 343HOPO was used here as a model case. It was not initially designed for separation applications and its performance should not be considered as the upper limit for a separation strategy that could be extended to an entire molecular family.

The selectivity of 343HOPO for tetravalent ions is so high that it is expected to form complexes even under very acidic conditions (experimentally observed^24^ in 3 M HCl for Sn^4+^), whereas it should release trivalent^25^ and divalent^24,26^ ions completely below pH~2 (Fig. 1). To our knowledge, no other reported class of ligands exhibits such behavior. The clear metal discrimination afforded by 343HOPO (bound M^4+^ versus free M^3+/2+/1+^) can therefore be leveraged as a chemical switch to isolate charged ions, a needed tool when separating Ac^3+^/Th^4+^ or Pu^4+^/Am^3+^ mixtures. If such an ultra-selective complexant is present in the aqueous phase, overall process selectivity is expected to be decoupled from extractant selectivity, since a system containing 343HOPO in the aqueous phase and a completely non-selective extractant in the organic phase will still result in highly efficient separation. Such ligand-driven performance enables more flexible solvent formulation and operational process conditions. It will also spare the cumbersome development of new extractants and can be applied to a variety of M^x+^/M^y+^ pairs, as demonstrated below.

### Actinium purification

**Fig. 2:**
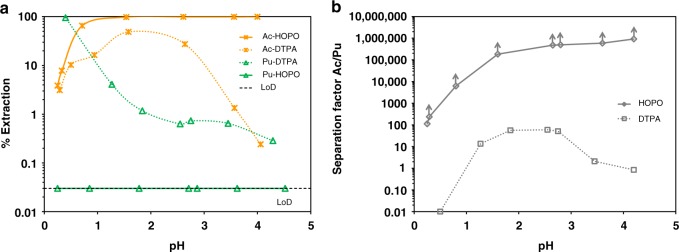
Separation of Ac from Pu. **a** Extraction yield of Ac^3+^ (stars) and Pu^4+^ (triangles) by 0.5 M HDEHP as a function of pH, in the presence of DTPA (dotted lines) or 343HOPO (solid lines). A logarithmic scale is used due to the low extraction yields of Pu^4+^. **b** Corresponding separation factors. Aqueous phase: 40 mM of chelator in sodium lactate/sodium nitrate buffer (*I* = 2 M). *V*_org_/*V*_aq_ = 1. *T* = 25 °C. LoD = Limit of detection. Data points with arrows are lower limits

**Fig. 3:**
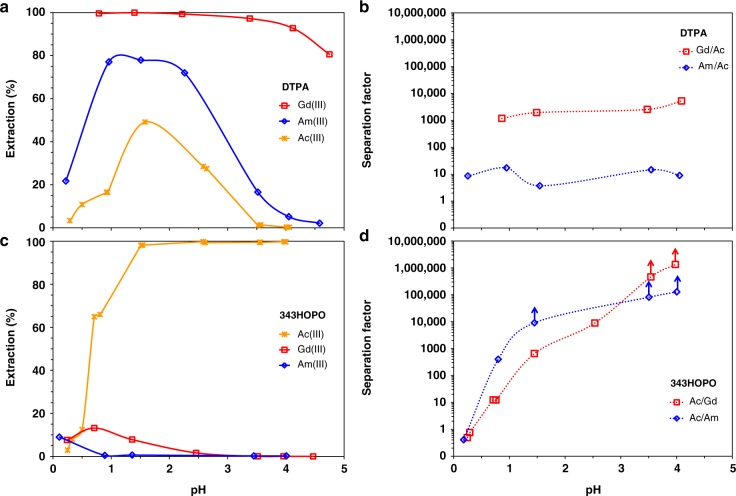
Separation of Ac from Gd and Am. Extraction yield of trivalent Ac (stars), Gd (squares), and Am (circles) by 0.5 M HDEHP as a function of pH, in the presence of DTPA (**a**) or 343HOPO (**c**). A logarithmic scale is used due to the low extraction yields of Pu^4+^. The corresponding separation factors in the presence of DTPA or 343HOPO are given in **b** and **d**, respectively. Aqueous phase: 40 mM of chelator in sodium lactate/sodium nitrate buffer (*I* = 2 M). *V*_org_/*V*_aq_ = 1, one contact. *T* = 25 °C. Data points with arrows are lower limits

While there is great interest in using ^225^Ac for targeted alpha therapy^27,28^, the development of ^225^Ac-based pharmaceuticals is still hindered by low isotope availability relative to potential market needs^1^. Furthermore, Ac chemistry is largely unexplored since (i) Ac^3+^ being the biggest trivalent ion of the periodic table^28,29^, there is no adequate surrogate to study its chemistry, and (ii) its highly-radioactive longest-lived isotope (^227^Ac, *t*_1/2_ = 21.8 year) is available in only μg amounts. Both ^227^Ac decay and ^225^Ac production yield mixtures of Ac^3+^, Th^4+^, Ra^2+^, and trivalent lanthanides. The ratio between unwanted elements and Ac is typically very high and purification options are limited^30^. 343HOPO exhibits very low affinity toward Ac^3+^ compared with other trivalent ions and has extremely high affinity for tetravalent ions, providing a straightforward tool to selectively isolate Ac. Figure 2 shows the Ac^3+^ and Pu^4+^ extraction yields by HDEHP (extractant widely used in hydrometallurgy, also known as D2EHPA)^31,32^ in the presence of 343HOPO at different pH values. Full Ac^3+^ extraction in the organic layer was achieved, while scavenging Pu^4+^ (a surrogate for Th^4+^) in the aqueous layer. Similar experiments in the presence of reference aqueous chelator DTPA, also used in the so-called TALSPEAK process^31^, showed partial extraction of both isotopes and no practical separation or recovery. After only a single step and despite a large initial Pu/Ac ratio (~10,000 mol/mol), SF_Ac/Pu_ values reached at least 1,000,000 in the presence of 343HOPO, combined with recovery yields of up to 99.70% for Ac in the organic phase and higher than 99.97% for Pu in the aqueous phase. In comparison, DTPA SF_Ac/Pu_ values were between 1 and 100, the typical selectivity range observed in hydrometallurgical processes, with less than 50% Ac recovery. Separation of Ac from trivalent impurities was also investigated. Figure 3 displays Ac^3+^, Am^3+^, and Gd^3+^ extraction profiles in both HDEHP/343HOPO-HNO_3_ and HDEHP/DTPA-HNO_3_ systems. Significant discrimination was observed for Ac^3+^ against Am^3+^ and Gd^3+^, with SF_Ac/Am_ and SF_Ac/Gd_ reaching 130,000 and 1,300,000, respectively. In the DTPA case, SF_Am/Ac_ and SF_Gd/Ac_ values were below 10 and 5300, respectively, and Ac recovery was low. A process flowsheet for ^225^Ac purification using the 343HOPO-HDEHP combination is proposed in Supplementary Fig. 3.

### Plutonium purification

Since the 1940’s, worldwide Pu inventory has evolved from almost 0 to ~2,500,000 kg due to anthropogenic activities^33,34^, and is estimated to increase by 70,000 kg/year^35^ based on civilian nuclear power generation forecasts. Pu materials must be properly safeguarded throughout their lifespan, which necessitates advanced nuclear forensic controls and reprocessing activities. In this context, isolation of Pu from minor actinide and fission product impurities is essential. The standard PUREX (Plutonium Uranium Redox EXtraction) liquid-liquid extraction process^36^ allows recovering and separating Pu and U from minor actinides and fission products. PUREX operates in concentrated HNO_3_ media and includes two critical steps: (i) UO_2_^2+^ and Pu^4+^ co-extraction into the organic phase (30 % TBP in diluent) while leaving trivalent ions in the aqueous phase, and (ii) reductive back-extraction of Pu as Pu^3+^ while leaving UO_2_^2+^ in the organic phase. The latter is indispensable for U/Pu separation because of TBP’s lack of selectivity between tetravalent and actinyl species. While reduction of Pu^4+^ to Pu^3+^ is achieved by addition of strong reducing agents into the liquid-liquid extraction batteries^37^, Pu^3+^ is inherently unstable, and this redox component creates constraints in the reactive TBP-nitric medium (reduction of HNO_3_ to HNO_2_ and formation of potentially explosive compounds such as HN_3_).

**Fig. 4:**
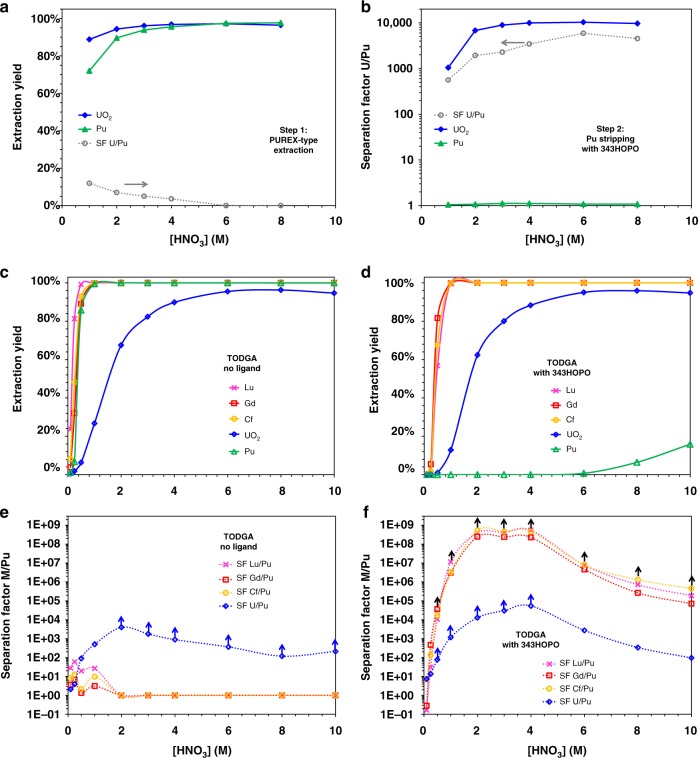
Separation of Pu from U, Gd, Lu, and Cf. Extraction of Pu^4+^ (triangles) and UO_2_^2+^ (diamonds) under typical PUREX conditions (**a**) and back-extraction in the presence of 1 mM 343HOPO (**b**). Dotted lines: separation factor U/Pu. Organic phase: 30 % TBP in kerosene. **c** and **d** show the extraction yields of Gd^3+^ (squares), Lu^3+^ (crosses), Cf^3+^ (circles), UO_2_^2+^, and Pu^4+^ by TODGA in the absence or presence of 1 mM 343HOPO, respectively. Corresponding separation factors are given in **e** and **f**. Organic phase: 0.1 M TODGA in kerosene. Aqueous solvent: HNO_3_. *T* = 25 °C. O/A = 1, one contact. Points with arrows are lower limits

A simple but effective modification of PUREX was investigated for non-reductive separation of Pu and U (Supplementary Fig. 4). Figure 4 shows that Pu^4+^ and UO_2_^2+^ extraction behaviors under typical PUREX conditions are expectedly very similar, with quantitative extraction of both elements and SF_U/Pu_ values below 3. After co-extraction of U and Pu by TBP, the unprecedented chelation properties of 343HOPO at high acidity and its charge-based selectivity can be leveraged to selectively strip Pu^4+^ from the organic phase. Efficient U/Pu separation was observed in the presence of 343HOPO (Fig. 4) by selective chelation of Pu^4+^, without significant interactions with UO_2_^2+^ over a broad range of acidity (up to 8 M HNO_3_) and SF_U/Pu_ values as high as 5800. The use of 343HOPO-type chelators could afford straightforward and efficient separation methods for the purification of U and Pu, and so, without using any redox-active chemical or non-volatile contaminant. This method also leads to very flexible process control, as demonstrated by the broad acidity range under which 343HOPO selectively scavenges Pu^4+^.

Another route, using TODGA as extractant, was explored for the purification of Pu not only from divalent ions but also from trivalent metals. TODGA is from the diglycolamide family used for resins separations^38,39^, and is currently investigated for next-generation nuclear waste treatment processes, such as EURO-GANEX^11,40,41^. TODGA is effective at extracting trivalent lanthanides and actinides from concentrated nitric media but its selectivity toward other ions, such as Pu^4+^, is very low. Very high yields were observed for the extraction of Gd^3+^, Lu^3+^, UO_2_^2+^, Pu^4+^, and Cf^3+^ from HNO_3_ (>99.5% if HNO_3_ > 1 M), emphasizing the lack of potential for practical separation (Fig. 4). In the presence of 343HOPO, Pu^4+^ is selectively held-back in the aqueous phase whereas the extraction behaviors of trivalent actinides, lanthanides, and uranyl are not impacted, offering a direct avenue for Pu recovery. SF values between the trivalent ions and Pu^4+^ are above 450,000,000 (LoD reached) whereas SF_U/Pu_ values are around 50,000. SF_U/Pu_ values are limited by the relatively low distribution factors of uranyl (D_U_ < 30) when using TODGA, as observed here and elsewhere^11^. Combining the high affinity of TODGA for trivalent lanthanides and actinides with the high affinity of TBP for uranyl and the selectivity of 343HOPO for Pu, a redox-free process was devised for the flash-recovery and purification of Pu from materials containing Pu, uranyl, minor actinides, and fission products (Supplementary Fig. 5).

### Berkelium purification

Bk and Cf are of particular interest due to their use as targets for the production of super-heavy elements, such as elements 117 (tennessine, named after a ^249^Bk target manufactured at Oak Ridge National Laboratory - ORNL, Tennessee)^42^ and 118 (oganesson, produced from ^249^Cf)^43^. ^252^Cf is also a strategic isotope for oil and gas exploration as well as quality control of nuclear reactors^1^. Bk and Cf are produced via prolonged neutron irradiation of Am/Cm targets, yielding mixtures of actinides from Am to Fm and some fission products^5^. These transplutonium elements have traditionally displayed very similar chemistries as they exhibit the +III oxidation state in solution and have almost identical ionic radii^44^. To separate Cf^3+^ from Bk^3+^, Bk^3+^ can eventually be oxidized to Bk^4+^ under harsh conditions (heating combined with excess NaBrO_3_ in 8 M HNO_3_) but it is unstable, which adds another level of complications to the eventual separation scheme. Campaigns conducted at ORNL for the purification of mg amounts of ^249^Bk take several months^5,45^ and result in relatively limited purification factors. The Bk isolation process^5^ comprises about 25 steps, with a purification factor for the entire procedure (product of SF values from all steps) of ~10^7^.

**Fig. 5:**
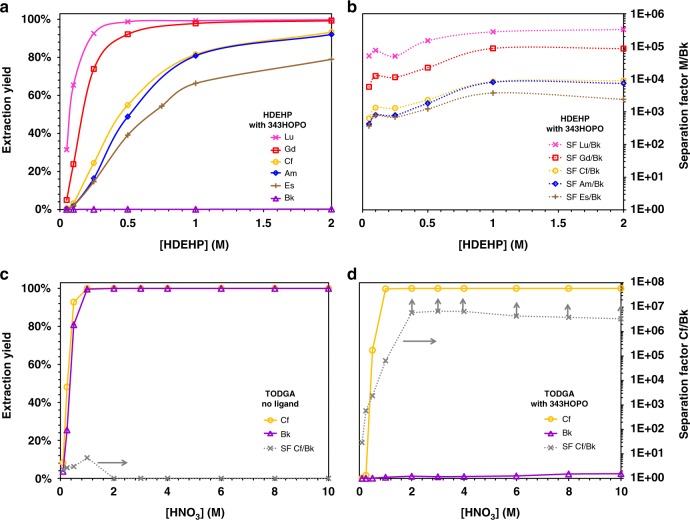
Separation of Bk, from Gd, Lu, Am, Cf, and Es. **a** Extraction of ^177^Lu (crosses), ^153^Gd (squares), ^243^Am (diamonds), ^249^Bk (triangles), ^249^Cf (circles) and ^253^Es (vertical crosses) by HDEHP as a function of the extractant concentration. Aqueous phase: 1 mM 343HOPO in 0.1 M HNO_3_ and 1.9 M NaNO_3_. Organic phase: HDEHP in kerosene. **b** Corresponding separation factors. A log scale is used for clarity. See Supplementary Fig. 8 for results as a function of the phase ratio. **c** and **d** show the extraction yields of ^249^Bk and ^249^Cf by TODGA (solid lines) and separation factors Cf/Bk (dotted lines) as a function of the acidity and in the absence or presence of 1 mM 343HOPO, respectively. Aqueous phase: 0 or 1 mM 343HOPO in HNO_3_. Organic phase: 0.1 M TODGA in kerosene. *T* = 25 °C. O/A = 1, one contact. Points with arrows are lower limits

343HOPO was recently shown^46^ to oxidize Bk^3+^ and stabilize Bk^4+^ in aqueous solution without addition of any redox-active species; a direct consequence of the ligand’s thermodynamic preference for tetravalent cations (*vide supra*). The separation strategy detailed above can therefore be applied to the isolation of Bk from all trivalent ions. Preliminary tests with extractant HDEHP at high acidity (0.1–6 M HNO_3_) show a drastic influence of 343HOPO on the Bk extraction by HDEHP (Supplementary Fig. 6). Comparisons with Pu^4+^, Gd^3+^, and Cf^3+^ confirm that Bk exists as Bk^3+^ in nitric media without chelator but forms a Bk^4+^ complex with 343HOPO even under very acidic conditions (Supplementary Fig. 7). Thus, isolation of Bk from its trivalent neighbors and lanthanides was studied in the HDEHP-HNO_3_−343HOPO system (Fig. 5). After a single step at room temperature, high extraction yields were observed for all tested trivalent ions, whereas Bk was selectively sequestered in the aqueous phase. SF_Bk/Lu_ values as high as 320,000 were obtained, and SF values between 3000 and 10,000 were reached between Bk and adjacent actinides Am^3+^, Cf^3+^, and Es^3+^. Similar tests in the presence of NTA, EDTA, CDTA, and DTPA showed no separation at all between Bk and Cf, (similar to what is observed without chelator) since this type of ligands is not strong enough to bind metal ions under acidic conditions and is not selective enough to oxidize Bk^3+^ to Bk^4+^. Importantly (Fig. 5 and Supplementary Fig. 8), the behavior of Bk in the presence of 343HOPO is completely decoupled from classic extraction parameters (extractant concentration, phase ratio), resulting in stable separation performance and robustness over a wide range of conditions. Given the system SF values, two consecutive steps would yield purification factors similar or higher than the current state-of-the-art process^5^.

Bk purification using TODGA as extractant was also investigated. TODGA is more relevant than HDEHP for the production of heavy actinides since it is effective at high acidity and is therefore compatible with the post-irradiation metallic target dissolution step. The extraction behavior of Bk in a TODGA-based system has never been reported^11^, but high extraction yields for Bk^3+^ could be expected based on the behavior of Cf^3+^. In nitric solutions without chelator, quantitative co-extraction of ^249^Bk^3+^ and ^249^Cf^3+^ by TODGA was observed, leading to no practical separation between the metals (Fig. 5). In stark contrast, addition of 343HOPO drastically changes the Bk extraction profile: less than 1% Bk is extracted throughout the acidity range whereas Cf extraction remains undisturbed, due to the non-interaction between trivalent ions and the aqueous chelator at high acidity. The two adjacent actinides can be separated over a very broad acidity range (0.5–10 M HNO_3_). Even if an unfavorable initial ratio Cf/Bk was used in those experiments (Cf/Bk~12,000), ^249^Bk samples completely exempt of ^249^Cf were obtained, notably so after only a single extraction. Experiments at various concentrations of extractant further confirmed the reliability of the TODGA-HNO_3_−343HOPO separation formulation (Supplementary Fig. 9) with SF_Cf/Bk_ > 1,000,000, regardless of the TODGA and HNO_3_ concentrations. Additional separation experiments with Gd^3+^ and Lu^3+^ further evidenced the reliability of this system for purifying Bk (Supplementary Fig. 10). Finally, the separation method was tested at the mCi level. Legacy samples of ^249^Bk/^249^Cf used in previous studies^44,47^ were gathered, evaporated, incinerated and reconstituted in 3 M HNO_3_ solutions containing multiple mCi of ^249^Bk with a Cf/Bk ratio of ~3.4 mol/mol. The solution was split in five, 343HOPO was added, and an extraction step was performed using TODGA. As detailed in Table 1, after only a single step and regardless of the conditions used, very efficient separation was observed with the recovery of essentially pure ^249^Bk in the aqueous phase (radiopurity > 99.999%, chemical purity > 99.8%) and high-purity ^249^Cf in the organic phase (chemical purity > 99.5%). These results further confirm the proposed strategy could be used with high-activity samples, and scaled up to either produce high-purity Bk isotopes or to remove Bk traces during Cm, Cf, Es, or Fm production. Subsequent recovery of all isotopes would be facilitated by incineration and reconstitution in the desired matrix.

**Table 1:** Separation of ^249^Bk and ^249^Cf at the mCi level using TODGA and 343HOPO^a^

Conditions	Phase	Radiopurity	Chemical purity
	Initial	Bk: 99.13 % ± 0.13 Cf: 0.87 % ± 0.03	Bk: 22.63 % ± 0.54 Cf: 77.37 % ± 0.54
O/A = 0.5 [343HOPO] = 1 mM	Organic	Bk: 52.70 % Cf: 47.30 %	Cf: 99.71 %
	Aqueous	Bk: > 99.999 %	Bk: > 99.8 %
O/A = 1.0 [343HOPO] = 1 mM	Organic	Bk: 64.85 % Cf: 35.15 %	Cf: 99.5 %
	Aqueous	Bk: > 99.999 %	Bk: > 99.8 %
O/A = 2.0 [343HOPO] = 1 mM	Organic	Bk: 70.60 % Cf: 29.40 %	Cf: 99.4 %
	Aqueous	Bk: > 99.999 %	Bk: > 99.8 %
O/A = 1.0 [343HOPO] = 5 mM	Organic	Bk: 79.72 % Cf: 20.28 %	Cf: 99.0 %
	Aqueous	Bk: > 99.999 %	Bk: > 99.8 %
O/A = 1.0 [343HOPO] = 25 mM	Organic	Bk: 84.94 % Cf: 15.06 %	Cf: 98.6 %
	Aqueous	Bk: > 99.999 %	Bk: > 99.8 %

^a^Separation conditions: 0.1 M TODGA in kerosene; 343HOPO in 3 M HNO_3_. T = 25 °C. One contact. Activity level: 2 mCi mL^−1^ (74 GBq L^−1^)

Noteworthy, the results presented above, although already providing better Cf/Bk separation than with any published method, do not represent optimum system performance since 343HOPO was not initially developed for separation applications and molecules with even higher selectivity could be designed. In addition, the three isotope purification examples examined in this work demonstrate the versatility of the proposed separation strategy, which could undoubtedly be transposed to other critical challenges^1^, such as those encountered with ^89^Zr^4+^/Y^3+^, ^177^Lu^3+^/Hf^4+^, ^134^La^3+^/^134^Ce^4+^, or ^117m^Sn^4+^/^116^Cd^2+^ purifications. We anticipate that the use of HOPO ligand derivatives as aqueous chelating hold-back reagents could pave the way to more reliable, flexible and efficient methodologies for metal cation separations.

## Methods

### Caution

The following isotopes were used in this work: ^153^Gd (ε, *t*_1/2_ = 240 days, 130 TBq/g), ^177^Lu (β^−^, *t*_1/2_ = 6.6 days, 4,100 TBq/g), ^225^Ac (α, *t*_1/2_ = 9.95 days, 2,100 TBq/g), ^233^U (α, *t*_1/2_ = 159200 years, 0.36 GBq/g), ^241^Pu (β^−^, *t*_1/2_ = 14.3 years, 3.8 TBq/g), ^242^Pu (α, *t*_1/2_ = 3.74 × 10^5^ years, 0.15 GBq/g), ^243^Am (α, *t*_1/2_ = 7388 year, 7.4 GBq/g), ^249^Bk (β^−^, *t*_1/2_ = 0.9 year, 61 TBq/g), ^249^Cf (α, *t*_1/2_ = 352 year, 0.15 TBq/g), and ^253^Es (α, *t*_1/2_ = 20.5 days, 932 TBq/g). All are highly radioactive, presenting serious health risks, and were manipulated in facilities specially designed for safe handling of long-lived radioactive materials at the Lawrence Berkeley National Laboratory (LBNL).

### Materials

^153^Gd(III) chloride and ^233^U(VI) nitrate were purchased from Eckert & Ziegler Isotope Products (Valencia, CA). ^177^Lu(III) chloride was purchased from Perkin Elmer Health Sciences (Shelton, CT). A Pu(IV) chloride stock solution containing a 50/50 mixture (Bq/Bq) of ^241^Pu and ^242^Pu and a stock solution of ^243^Am(III), prepared by dissolution of ^243^Am_2_O_3_ in 1 M HNO_3_, were from LBNL inventory. ^225^Ac(III), ^249^Bk(III), and ^249^Cf(III) were obtained as chlorides from ORNL. A ^253^Es(III) perchlorate solid sample was provided by Prof. J. Shafer (Colorado School of Mines). 343HOPO was from LBNL inventory^14^. Standard solutions of 0.1 M and 6.0 M HNO_3_ (BDH VWR Analytical), 70% HNO_3_ (Sigma Aldrich), NaNO_3_ (>99%, ACS grade, VWR), sodium lactate (Sigma Aldrich), TODGA (>99%, Technocomm Ltd.), HDEHP (>95%, Merck), tributyl phosphate (TBP, >98%, Alpha Aesar), kerosene low odor (Alpha Aesar), DTPA (>98%, TCI), and Ultima Gold LLT (Perkin Elmer) were used as received. All solutions were prepared using deionized water purified by a Millipore Milli-Q reverse osmosis cartridge system. Stocks were stored at 8 °C in the dark between experiments.

### Methods

pH measurements were performed with a glass electrode (Metrohm - *Micro Combi* - response to [H^+^]) calibrated at 25.0 °C using three NIST standards. Extraction samples were analyzed by liquid scintillation counting (LSC). The distribution coefficient, *D*(M), of a given metal, M, is defined in Eq. (1), where [M]_organic_ and [M]_aqueous_ are the respective total concentrations of M in the organic and aqueous layers, after extraction. Both concentrations are proportional to the volumetric activity (in Bq L^−1^) determined by LSC. The separation factor, SF_M1/M2_, between two metals, M_1_ and M_2_, and extraction yield were calculated according to Eq. (2) and Eq. (3), respectively.

1 $$D\left( {\mathrm{M}} \right) = \frac{{\left[ {\mathrm{M}} \right]_{{\mathrm{organic}}}}}{{\left[ {\mathrm{M}} \right]_{{\mathrm{aqueous}}}}} = \frac{{[{\mathrm{Activity}}]_{{\mathrm{organic}}}}}{{[{\mathrm{Activity}}]_{{\mathrm{aqueous}}}}}$$

2 $${\mathrm{SF}}_{{\mathrm{M}}1/{\mathrm{M}}2} = \frac{{D({\mathrm{M}}_1)}}{{D({\mathrm{M}}_2)}}$$

3 $$\% {\mathrm{Extraction}} = \frac{{[{\mathrm{Activity}}]_{{\mathrm{organic}}} \times V_{{\mathrm{organic}}}}}{{[{\mathrm{Activity}}]_{{\mathrm{organic}}} \times V_{{\mathrm{organic}}} + [{\mathrm{Activity}}]_{{\mathrm{aqueous}}} \times V_{{\mathrm{aqueous}}}}} \times 100$$

In a typical experiment, the solvent (extractant diluted in kerosene) was pre-conditioned by shaking one volume of solvent with three volumes of aqueous phase (typically HNO_3_) at room temperature for 1 h, thrice. For radioisotope extractions, at least 400 µL aqueous phase (typically containing HNO_3_, a chelator and a radioisotope) and solvent were placed in air-tight screw-capped plastic tubes, triply-contained, and shaken in a thermoshaker at 300 rpm, 25 °C, for at least 30 min. Samples were then centrifuged for 5 min at 3000 rpm and phases separated before analysis. LSC analyses were performed on a Packard Tri-Carb B4430 instrument (Perkin Elmer) after mixing sample aliquots or sample dilution aliquots (10–200 µL) with 10 mL of scintillation cocktail (UltimaGold LLT). Samples were counted for at least 6 min and results were background subtracted. The analytical samples contained between 0 and 800,000 CPM. Mixtures of ^249^Bk and ^249^Cf were analyzed using α/β discrimination and energy windows were set to 0–50 keV for low-energy β particles of ^249^Bk and 50–1000 keV for α particles of ^249^Cf. ^243^Am and ^225^Ac samples were counted at secular equilibrium.

## Supplementary information

Supplementary Information

## Data Availability

All data generated or analysed during this study are included in this published article (and its supplementary information files).
