# Supplementary material for: Ultra-selective ligand-driven separation of strategic actinides
Source: Nat Commun. 2019 Jun 4;10:2438. doi: 10.1038/s41467-019-10240-x (PMC6547845; doi:10.1038/s41467-019-10240-x)
Supplement: Supplementary file 1 — Supplementary Information [file 41467_2019_10240_MOESM1_ESM.pdf]

Ultra-Selective Ligand-Driven Separation of Strategic Actinides

Deblonde et al.

Supplementary Information

Supplementary Tables

Supplementary Table 1. List of ligands and extractants mentioned in this study.

**TBP**<br>(n-tributyl phosphate)<br>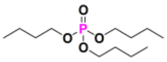	**DOTA**<br>(1,4,7,10-Tetraazacyclododecane-1,4,7,10-tetraacetic acid)<br>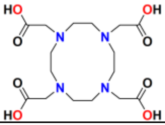	**CDTA**<br>(Trans-1,2-cyclohexanediamine-N,N,N′,N′-tetraacetic acid)<br>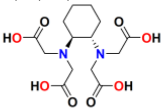
**HDEHP**<br>**(**Bis(2-ethylhexyl) phosphate, also D2EHPA or DEHPA)<br>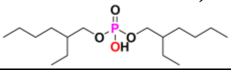	**DPA**<br>(Dipicolinic acid)<br>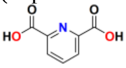	**Citric**<br>Citric acid)<br>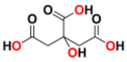
**TODGA**<br>(N,N,N′,N′-tetraoctyl diglycolamide)<br>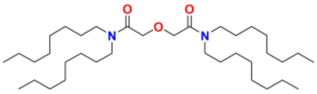	**DTPA**<br>(Diethylenetriaminepentaacetic acid)<br>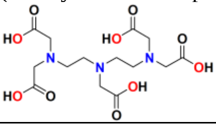	**DGA**<br>(Diglycolic acid)<br>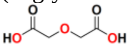
**343HOPO**<br>(3,4,3-LI(1,2-HOPO))<br>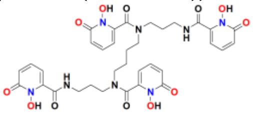	**EDTA**<br>(Ethylenediaminetetraacetic acid)<br>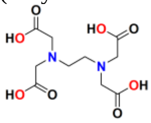	**NTA**<br>(Nitrilotriacetic acid)<br>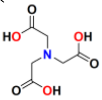
**343CAM**<br>(3,4,3-LI-CAM)<br>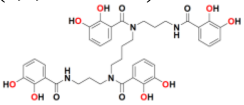	**EEDTA**<br>(2,2',2",2"'-[Oxybis(2,1-ethanediyl nitrilo)]tetraacetic acid, also BAETA)<br>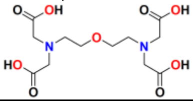	**TAM-macrocycle**<br>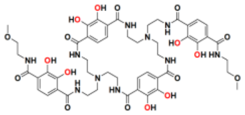
**Bis-TREN-Me-3,2-HOPO**<br>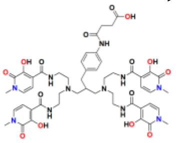	**EGTA**<br>(Ethylene glycol tetraacetic acid)<br>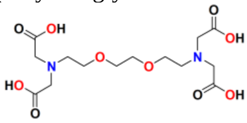	**TTHA**<br>(Triethylenetetramine-N,N,N',N",N"',N"'-hexaacetic acid)<br>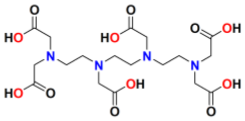
**5LIO-Me-3,2-HOPO**<br>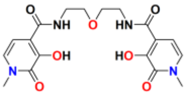	**Me-EDTA**<br>(Propylenediamine-N,N,N′,N′-tetraacetic acid, also PDTA)<br>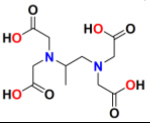	**TEDTA**<br>(2,2',2",2"'-[Sulfanediylbis(2,1-ethanediylnitrilo)]tetraacetic acid)<br>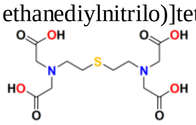
**5LI-Me-3,2-HOPO**<br>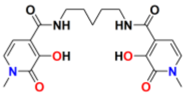	**HEDTA**<br>(Hydroxyethylethylenediaminetriacetic acid)<br>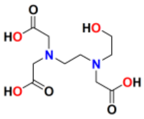	**TGA**<br>(Thioglycolic acid)<br>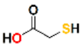
**Acetic**<br>(Acetic acid)<br>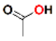	**IDA**<br>(Iminodiacetic acid)<br>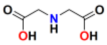	**TDA**<br>(Thiodiacetic acid)<br>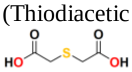

Supplementary Figures

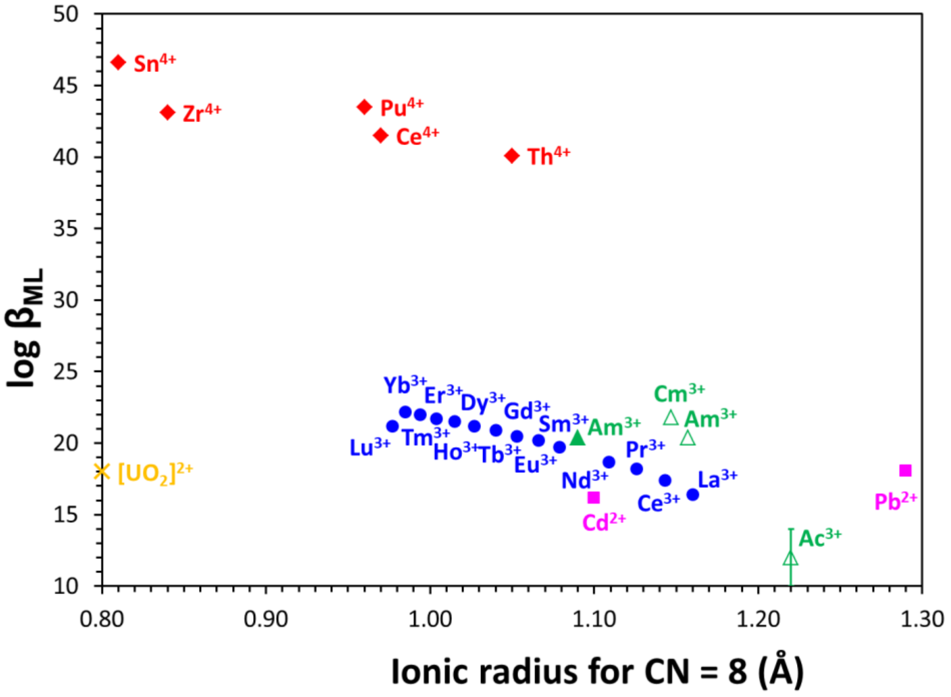

Supplementary Figure 1. Stability constants of the complexes of 343HOPO against the ionic radius of the metal ions (for a coordination number, CN, of 8). Ionic radius values were taken from Shannon (1976) and Lundberg and Persson (2016). Since no ionic radius value for a CN of 8 has been published for Ac^{3+}, and Cm^{3+}, ionic radius values for a CN of 9 were used and plotted for comparison purposes (empty symbols). The uranyl ion is arbitrary placed at 0.8 Å for comparison.

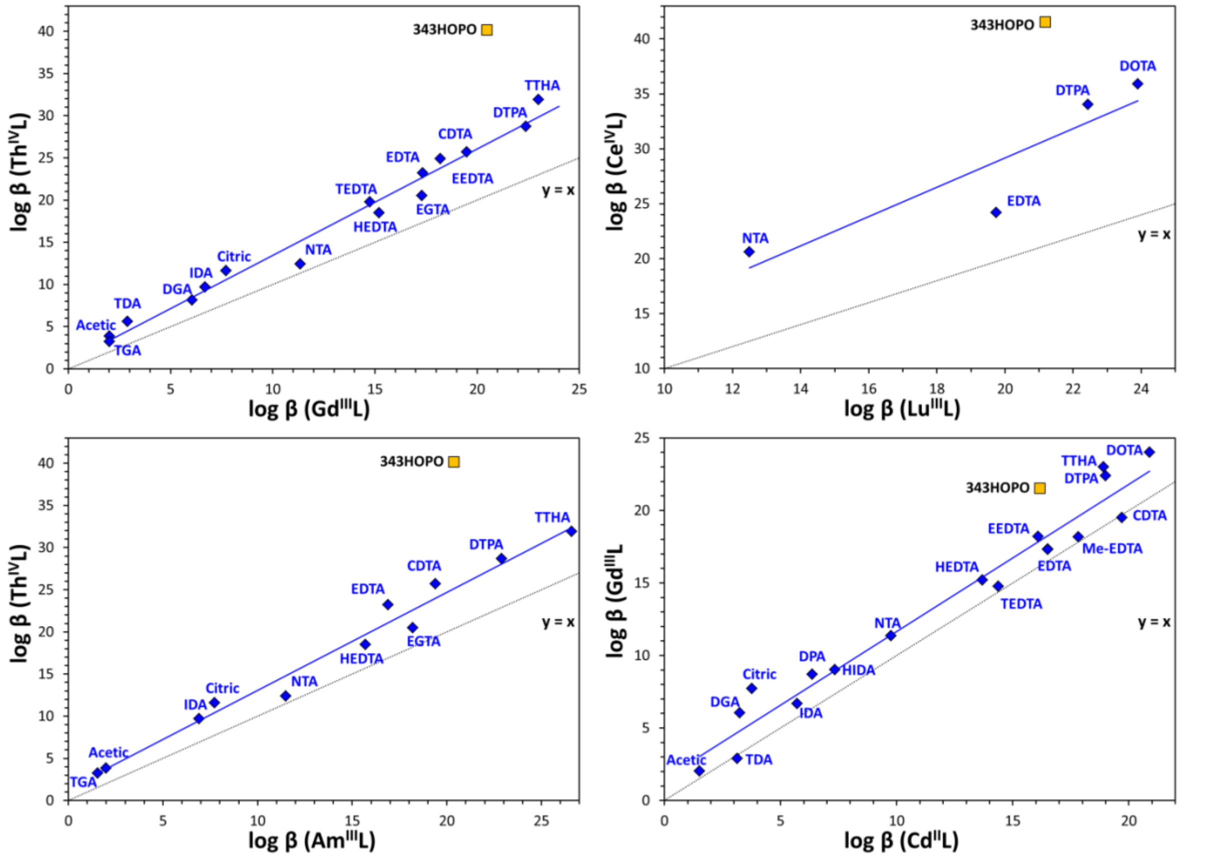

Supplementary Figure 2. Additional comparisons of ligand selectivity between Th^{4+} and Gd^{3+}, Ce^{4+} and Lu^{4+}, Th^{4+} and Am^{3+} as well as Gd^{3+} and Cd^{2+}. See Table S1 for the full names of the ligands. The line y = x corresponds to no selectivity. Log β values were taken from the National Institute of Standards and Technologies database (NIST46 - NIST Critically Selected Stability Constants of Metal Complexes: Version 8.0).

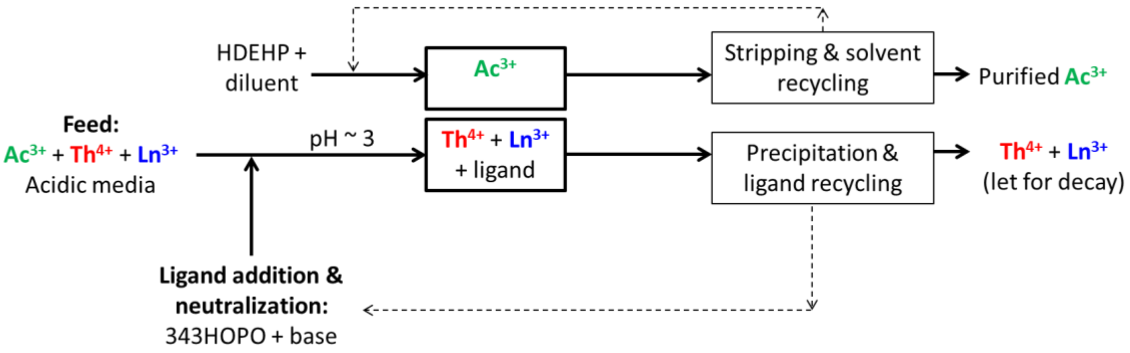

Supplementary Figure 3. General process flowsheet proposed for the purification of actinium isotopes using 343HOPO and HDEHP.

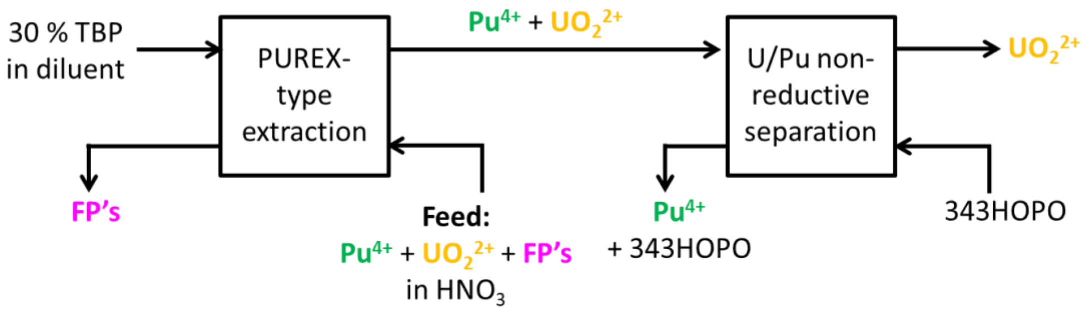

Supplementary Figure 4. Conceptual flowsheet for the modification of the PUREX method allowing the recovery and separation of uranyl and Pu^{4+} ions without using a reductive striping step for Pu. FP = Fission product.

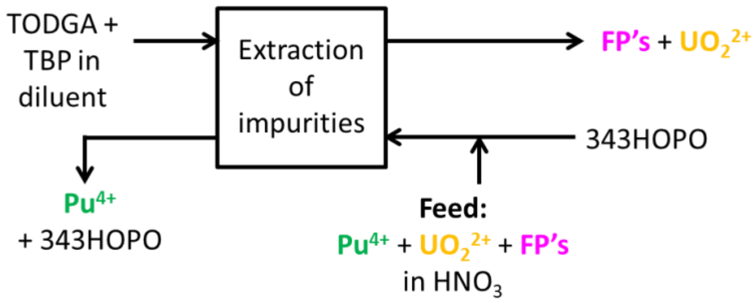

Supplementary Figure 5. Proposed flowsheet for the flash and redox-free purification of Pu from uranyl and fission products (FP).

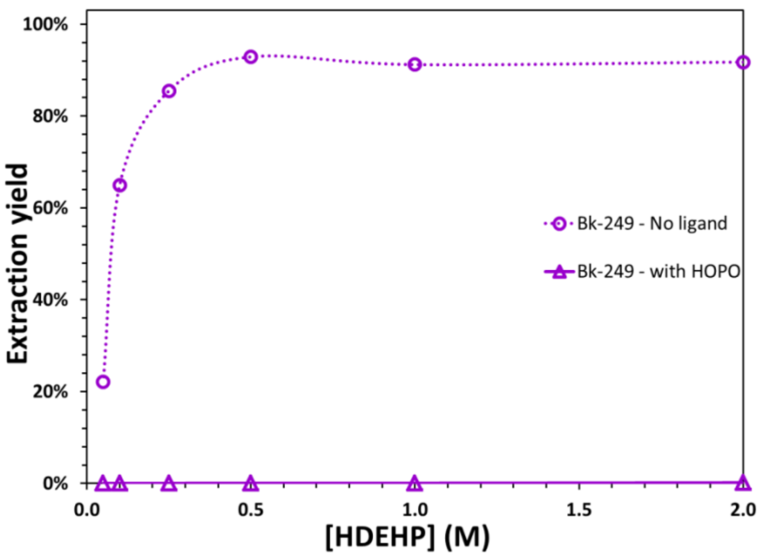

Supplementary Figure 6. Extraction profile of ^{249}Bk by HDEHP in the absence (dotted line) or presence (solid line) of 343HOPO. Aqueous phase: 0 or 1 mM 343HOPO in 0.1 M HNO_3 and 1.9 M $NaNO_3$. Organic phase: HDEHP in kerosene. O/A = 1. T = 25°C. One contact.

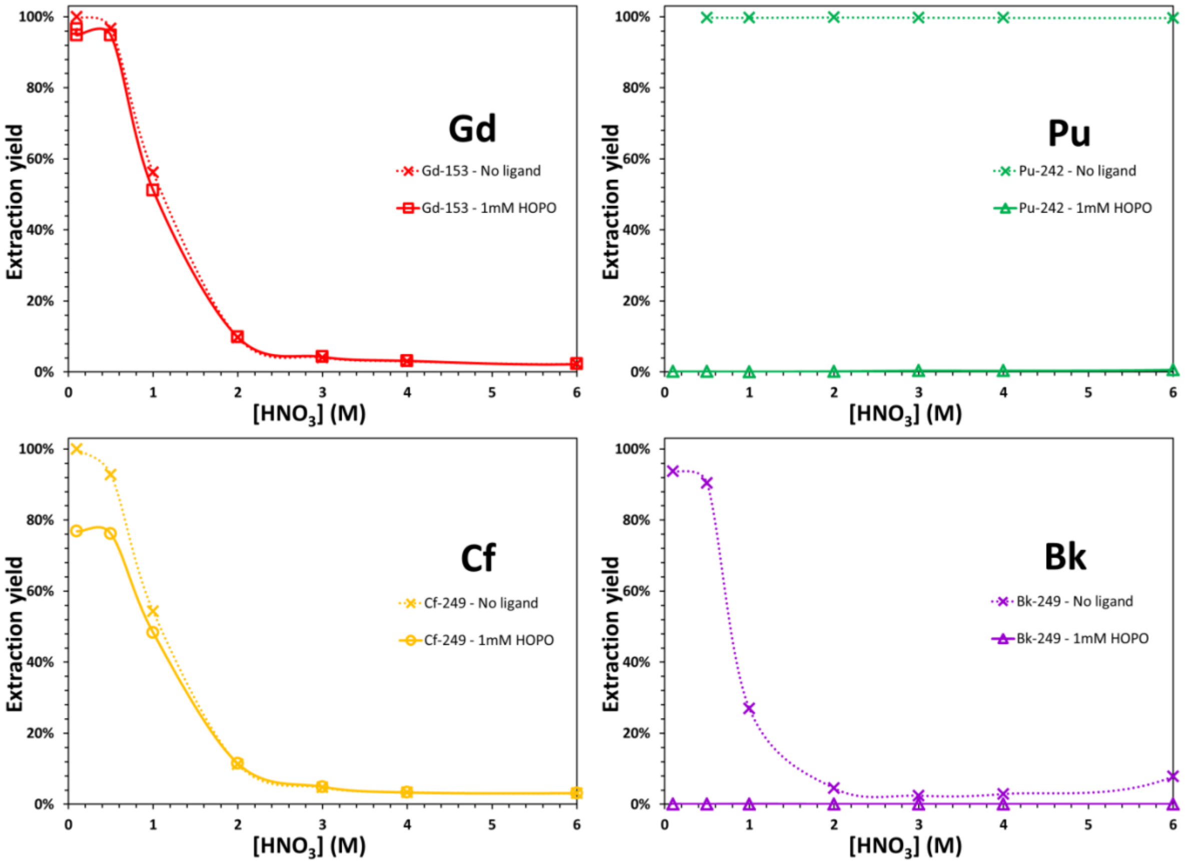

Supplementary Figure 7. Extraction profile of ^{153}Gd, ^{242}Pu, ^{249}Bk, and ^{249}Cf by HDEHP as a function of the acidity and in the absence (dotted line) or presence (solid line) of 343HOPO. Aqueous phase: 0 or 1 mM 343HOPO in HNO_3 (0.1 to 6 M). Organic phase: 0.75 M HDEHP in kerosene. O/A = 1. T = 25°C. One contact.

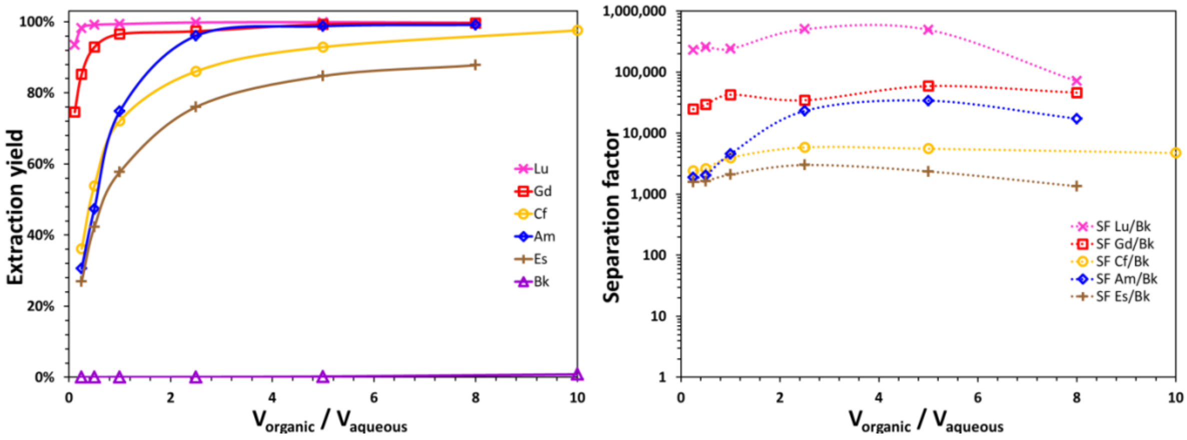

Supplementary Figure 8. Left: Extraction profiles of ^{177}Lu (crosses), ^{153}Gd (squares), ^{243}Am (diamonds), ^{249}Bk (triangles), and ^{253}Es (vertical crosses) as a function of the volume phase ratio. **Right:** Corresponding separation factors. Aqueous phase: 1 mM 343HOPO in 0.1 M HNO_3 and 1.9 M $NaNO_3$. Organic phase: 0.75 M HDEHP in kerosene. T = 25°C. One contact. See Fig. 5 for corresponding data as a function of the extractant concentration.

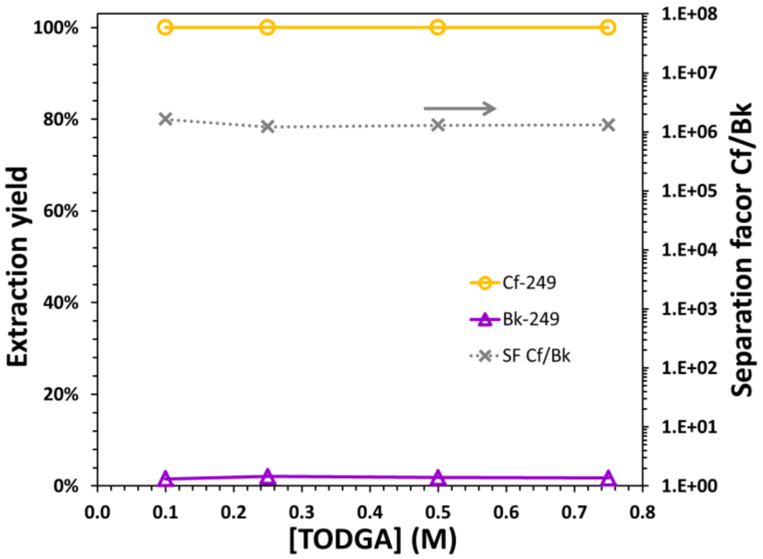

Supplementary Figure 9. Extraction profiles of ^{249}Bk (triangles), and ^{229}Cf (circle) and corresponding separation factors (dotted line) as a function of the extractant concentration. Aqueous phase: 1 mM 343HOPO in 3 M HNO_3. Organic phase: TODGA in kerosene. O/A = 1. One contact. T = 25°C. Separation factor values are lower limits due to the quantitative extraction of Cf under these experimental conditions.

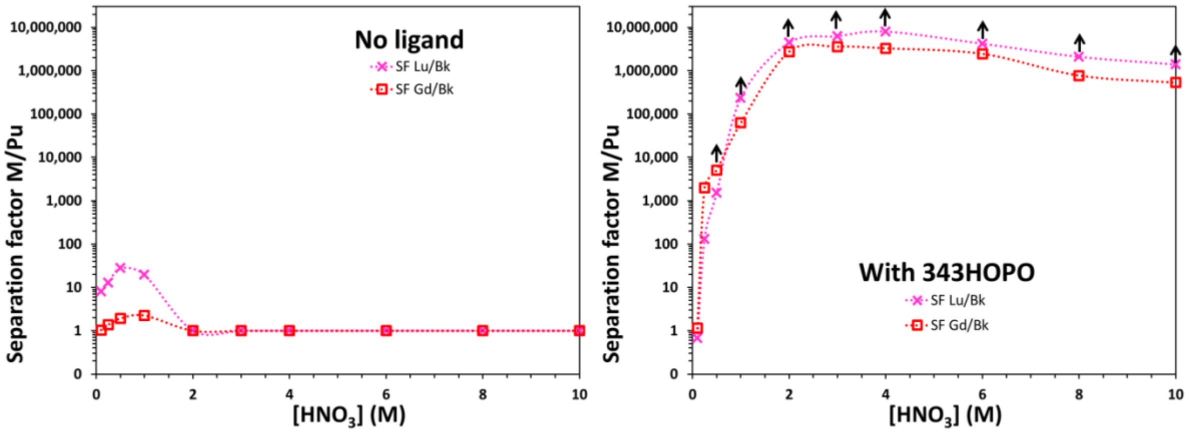

Supplementary Figure 10. Separation factors Lu/Bk and Gd/Bk obtained with the TODGA/343HOPO-HNO_3 extraction system. Aqueous phase: 1 mM 343HOPO in HNO_3. Organic phase: 0.1 M TODGA in kerosene. O/A = 1. One contact. T = 25°C. Arrows indicate lower limit due to either the total extraction of Lu^{3+} and Gd^{3+} or the total scavenging of Bk in the aqueous phase.
